# Incidence of eye problems in children aged 3–6 years during and after the COVID-19 pandemic

**DOI:** 10.3389/fmed.2025.1620391

**Published:** 2026-01-12

**Authors:** Peng Ye, Ying Zhang, Yunrong Fu, Peiling Cai, Yu Xie, Xinwei Chen, Mengping Wang, Mingyue Zhang, Ronglian Guo

**Affiliations:** 1 School of Preclinical Medicine, Chengdu University, Chengdu, Sichuan, China; 2 Jinniu Maternity and Child Health Hospital of Chengdu, Chengdu, Sichuan, China; 3 Clinical Medical College & Affiliated Hospital of Chengdu University, Chengdu, Sichuan, China; 4 Department of Pediatrics, Zhongshan Hospital of Xiamen University, School of Medicine, Xiamen University, Xiamen, Fujian, China

**Keywords:** anisometropia, astigmatism, COVID-19, hyperopia, myopia, pre-school children

## Abstract

**Background:**

Incidence of eye problems increased during the COVID-19 pandemic when home confinement measures were adopted. The aim of this study was to investigate the changes of incidence of eye problems after the ending of COVID-19 pandemic.

**Methods:**

Vision screening results were retrospectively extracted from the health examination results of children from Southwestern China who were aged 3–6 years in either 2022 or 2023. After interpretation of the vision screening results using an official standard, percentages of abnormal vision screening results were calculated and compared between year 2022 and 2023 using statistical software.

**Results:**

In all, vision screening results were extracted from 26396 children in 2022 and 31324 children in 2023. The standardized overall incidence of eye problems was 9.13% in 2022, and 8.78% in 2023. The standardized incidence of myopia, hyperopia, astigmatism, and anisometropia were 0.84%, 0.55%, 8.64%, and 0.87% in 2022, and 0.60%, 0.61%, 8.06%, 0.45% in 2023, respectively. Logistic regression showed that odds of having eye problems, myopia, astigmatism, or anisometropia were significantly lower in 2023.

**Conclusion:**

In the cohort of children aged 3–6 years, the overall incidence of eye problems and incidence of myopia, astigmatism and anisometropia was lower in 2023, together with higher incidence of hyperopia in 2023. Incidence of astigmatism was much higher than that of other types of eye problems. More efforts should be made to lower the incidence of astigmatism in pre-school children, as well as understand the underlying mechanism on the impact of COVID-19 on the eye health of pre-school children.

## 1 Introduction

On 11 March, 2020, the World Health Organization (WHO) designated coronavirus disease 2019 (COVID-19) as a pandemic (1). During the COVID-19 pandemic, in order to slow down the spreading of the disease, home confinement measures have been adopted in many countries, which started in 2020. During that period, many researchers observed increased incidence of eye problems (e.g., myopia, astigmatism, or anisometropia) in children compared to pre-pandemic period, possibly due to limited outdoor activities and prolonged screen time during the home confinements (2–12). As summarized in two relevant systemic reviews, myopia progression appeared to be accelerated during COVID-19 pandemic, possibly due to reduced outdoor time and increased screen time (13, 14). In addition, incidence of astigmatism has been found to be significantly increased in children during COVID-19 pandemic (10, 11). Besides of the above studies which were focusing on school-aged children, two other studies investigated the incidence of myopia in pre-school children (15, 16). Both studies reported relatively stable myopia rate during the COVID-19 pandemic compared to pre-pandemic period, which indicated relatively smaller impact of home confinement on the myopia progression in pre-school children.

During the COVID-19 pandemic, when spreading of the disease was controlled, home confinement measures were temporarily suspended, and most social activities were restored (e.g., year 2021 in China). Previous studies also investigated the myopia rates of children during this period (17, 18). One study reported that mean spherical equivalent refraction of school-aged children returned to pre-pandemic level in 2021 (18). The other study showed conflicting results that the impact of home confinement on myopia development did not vanish and could still be observed in 2021 (17). For pre-school children, study by Li et al. (16) reported that their myopia rate slightly increased from 3.3% in 2020 to 3.5% in 2021.

On 5 May 2023, WHO announced that COVID-19 was no longer a public health emergency of international concern (1), putting an end to the 3-years pandemic. All the home confinement measures were removed and social activities were fully restored. Although increased incidence of eye problems in children was observed during the COVID-19 pandemic, few studies compared the incidence of myopia or astigmatism in children before and after the ending of COVID-19 pandemic, especially in pre-school children. It is still unknown whether the impact of COVID-19 on the eye health of children has also decreased or vanished after all the hurdles for social activities were removed.

In this study, we aimed to compare the incidence of eye problems (hyperopia, myopia, astigmatism, and anisometropia) in children aged 3–6 years during and after COVID-19 pandemic period. Results of this study should be treated carefully since the vision screening of this study was performed using an autorefractor system which was known to overestimate myopia and underestimate hyperopia (19–21).

## 2 Materials and methods

Vision screening results were retrospectively and anonymously collected from the results of yearly obligatory health examination for pre-school children provided by Jinniu Maternity and Child Health Hospital of Chengdu. The health examination was obligatory for the pre-school children, including those with ocular disease or previous treatments. During the data extraction, vision screening results and demographic information were only collected from children who were 3 to 6 years old in either 2022 or 2023. Informed consent was waived by the Institutional Review Board of Affiliated Hospital of Chengdu University since this was a retrospective and non-interventional study.

The vision screening as included in the routine health examination for pre-school children was performed using a commercial vision screener (VS100, Welch Allyn, USA). The measurement was non-cycloplegic. One reading per eye was usually taken, but in case of uncertain results, multiple measurements were performed to ensure the correctness of the results. To ensure the quality of measurement results, vision screener was regularly maintained and calibrated. The readouts of the vision screener were recorded and interpreted following the Standard of Eye Health and Vision Examination Services for Children Aged 0 ∼ 6 years released by National Health Commission of the People’s Republic of China. The vision screening results were labeled abnormal if the following criteria were met. For children aged 3 ∼ 4 years: astigmatism > 2.00 D; hyperopia > +4.00 D; myopia <−3.00 D; anisometropia: differences in hyperopia or myopia > 1.50 D, or difference in astigmatism > 1.00 D. For children aged 5 ∼ 6 years: astigmatism > 1.50 D; hyperopia > +3.50 D; myopia <−1.50 D; anisometropia: differences in hyperopia or myopia > 1.50 D, or difference in astigmatism > 1.00 D. If abnormal vision screening results were found in left and/or right eye, the case was suspected to have eye problems, including myopia, hyperopia, astigmatism, and/or anisometropia. Children with multiple abnormal vision screening results (e.g., both myopia and astigmatism) were counted once in the calculation of the overall prevalence of eye problems. Demographic information was also collected during the health examination, including gender, date of birth, and date of health examination.

The extracted vision screening results and demographic information of pre-school children were firstly analyzed using Microsoft Excel (Microsoft Corporation). Incidence of eye problems were calculated by dividing the number of cases suspected to have eye problems in either 2022 or 2023 by the total number of children in the same year who were involved in this study, including the specific incidence of myopia, hyperopia, astigmatism, and anisometropia. Age on the day of vision screening was calculated by subtracting date of birth from the date of health examination, and the results were rounded down to the nearest integer (e.g., children aged 3 years 0 day to 3 years 11 months 30 days were all considered 3 years old). Incidence of eye problems, including myopia, hyperopia, astigmatism, and anisometropia, was compared between year 2022 and 2023 using Chi-square test. Subgroup analysis was also performed after stratification by gender (male and female) and age group (3-years old, 4-years old, 5-years old, and 6-years old). All the statistical analysis was performed using IBM SPSS 19.0 (IBM), and *P* < 0.05 was considered statistically significant.

## 3 Results

As shown in Table 1, in all, vision screening results were retrospectively collected from the health examination results of 57720 children in 2022 (*n* = 26396) and 2023 (*n* = 31324) (Supplementary Table 1). No significant difference was found in gender between 2022 (percentage of male: 51.9%) and 2023 (percentage of male: 52.1%, *P* = 0.615). Significant difference was found in the age distribution between 2022 and 2023 (*P* < 0.001). Higher percentages of age 4 (31.5%) and 5 (36.8%) was observed in 2022 compared to 2023 (29.5% and 30.9%, respectively), and higher percentages of age 3 (14.5%) and 6 (25.1%) were shown in 2023 compared to 2022 (12.1% and 19.6%, respectively). The time of vision screening was similar between 2022 and 2023, which scattered from March to July in both years. After dividing the vision screening time into seasons (spring: March to May; summer: June to July), higher proportion of children attended vision screening in spring in 2023, compared to 2022 (76.7% versus 23.3%, respectively, *P* < 0.001). Although the socioeconomic status of the children was not recorded, the average socioeconomic status of the children included in this study should be similar between 2022 and 2023, considering that the two cohorts were from the region, attended the same group of kindergartens, and were consisted of large numbers of children. Length of outdoor activities of the children was also not recorded since this was not the main focus of this study. However, outdoor activities were expected to be decreased during the pandemic (in 2022) when home confinement was often conducted. Subgroup analysis by gender, age or vision screening time was performed and the results are described below.

**TABLE 1:** Demographic information.

Demographics	Overall	2022	2023	*P*
*N*	57720	26396	31324	
**Gender**				
Male	52.0%	51.9%	52.1%	0.615
Female	48.0%	48.1%	47.9%	
**Age**				
3 years old	13.4%	12.1%	14.5%	<0.001
4 years old	30.4%	31.5%	29.5%	
5 years old	33.6%	36.8%	30.9%	
6 years old	22.6%	19.6%	25.1%	
**Vision screening time**				
Spring (March - May)	65.8%	52.9%	76.7%	<0.001
Summer (June - July)	34.2%	47.1%	23.3%	

**FIGURE 1:**
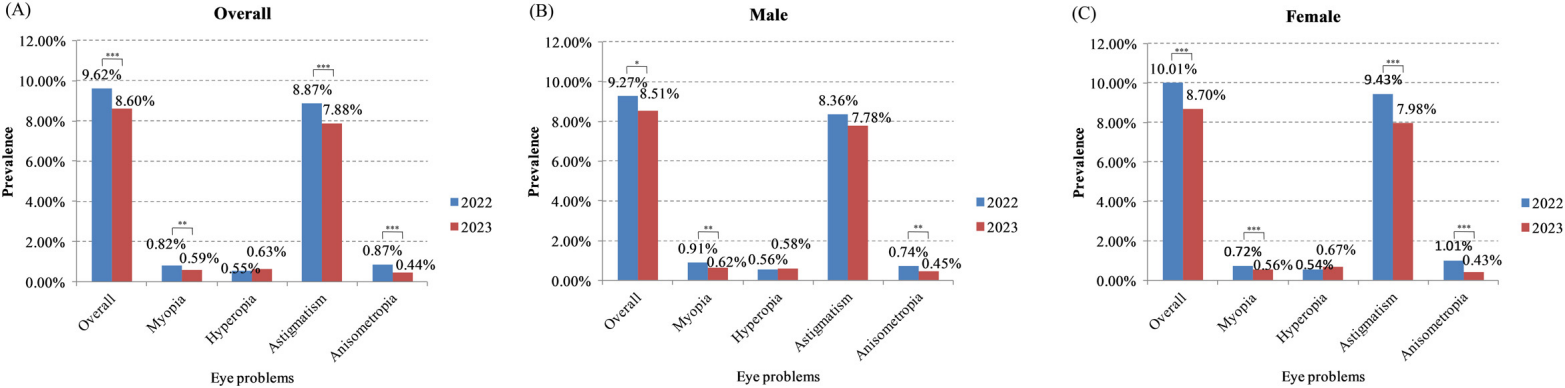
Prevalence of myopia, hyperopia, astigmatism, and anisometropia in the study cohort during and after the COVID-19 pandemic. **(A)** Overall prevalence; **(B)** prevalence of eye problems in male children; **(C)** prevalence of eye problems in female children. *P < 0.05, **P < 0.01, ***P < 0.001.

**FIGURE 2:**
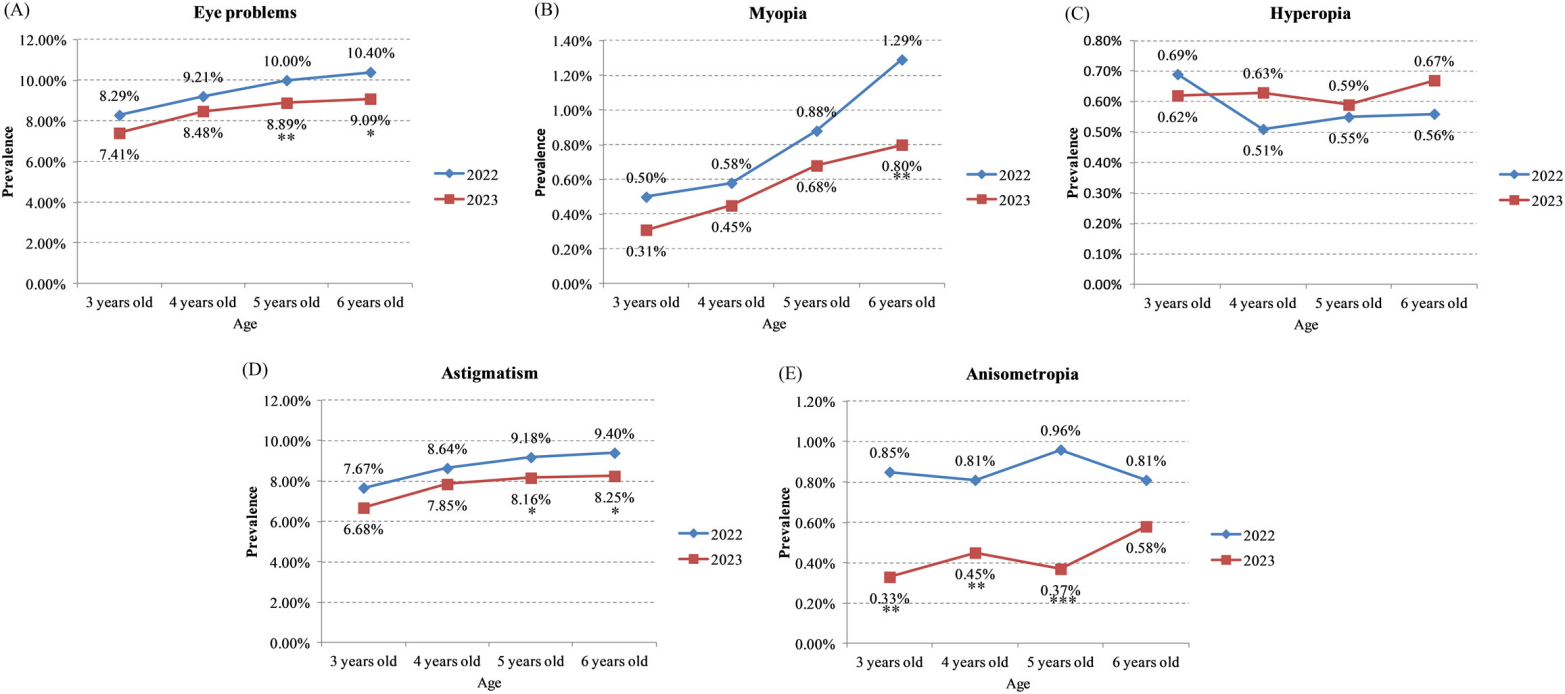
Prevalence of eye problems in children of different age during and after the COVID-19 pandemic. **(A)** Overall prevalence; **(B)** prevalence of myopia; **(C)** prevalence of hyperopia; **(D)** prevalence of astigmatism; **(E)** prevalence of anisometropia. *P < 0.05, **P < 0.01, ***P < 0.001.

As shown in Figure 1 and Table 2, the overall incidence of eye problems was 9.62% in 2022, and 8.60% in 2023 (*P* < 0.001). After standardization by age and vision screening time, the difference of overall incidence of eye problems was smaller between 2022 and 2023 (9.13% in 2022 versus 8.78% in 2023). After stratification by gender, the overall incidence of eye problems was significantly lower in 2023 in both male (*P* = 0.022) and female children (*P* < 0.001). After stratification by age, incidence of eye problems were also lower in 2023 in all age groups, although the difference was not significant in children aged 3 or 4 years (3-years old: *P* = 0.151; 4-years old: *P* = 0.089; 5-years old: *P* = 0.008; 6-years old: *P* = 0.013; Figure 2A and Table 2). After stratification by vision screening time, the overall incidence of eye problems were lower in 2023 in both spring and summer, but the differences were not statistically significant (*P* = 0.063 and 0.099, respectively, Table 3). Logistic regression analysis showed that the odds of having eye problems was significantly larger when age increases (adjusted odds ratio = 1.070, *P* < 0.001) or when vision screening was performed in summer (adjusted odds ratio = 1.230, *P* < 0.001, Table 4). Odds of having eye problems were significantly smaller in 2023 (adjusted odds ratio = 0.928, *P* = 0.012, Table 4).

**TABLE 2:** Comparison of the prevalence of eye problems between 2022 and 2023, % (95% confidence interval).

	Overall		Gender		Age			
Year	Crude	Standardized (age and vision screening time)	Male	Female	3 years old	4 years old	5 years old	6 years old
**Eye problems**								
2022	9.62 (9.27–9.98)	9.13 (8.78–9.48)	9.27 (8.78–9.75)	10.01 (9.48–10.53)	8.29 (7.34–9.25)	9.21 (8.59–9.83)	10.00 (9.40–10.59)	10.40 (9.57–11.23)
2023	8.60 (8.29–8.91)	8.78 (8.47–9.10)	8.51 (8.09–8.94)	8.70 (8.25–9.15)	7.41 (6.65–8.17)	8.48 (7.91–9.05)	8.89 (8.32–9.45)	9.09 (8.46–9.73)
*P*	<0.001		0.022	<0.001	0.151	0.089	0.008	0.013
**Myopia**								
2022	0.82 (0.71–0.93)	0.84 (0.73–0.95)	0.91 (0.75–1.07)	0.72 (0.57–0.86)	0.50 (0.26–0.75)	0.58 (0.41–0.74)	0.88 (0.69–1.06)	1.29 (0.99–1.60)
2023	0.59 (0.51–0.68)	0.60 (0.52–0.69)	0.62 (0.50–0.74)	0.56 (0.44–0.68)	0.31 (0.15–0.47)	0.45 (0.32–0.59)	0.68 (0.52–0.85)	0.80 (0.60–1.00)
*P*	0.001		0.003	<0.001	0.178	0.255	0.126	0.006
**Hyperopia**								
2022	0.55 (0.46–0.64)	0.55 (0.46–0.63)	0.56 (0.44–0.69)	0.54 (0.42–0.67)	0.69 (0.40–0.98)	0.51 (0.35–0.66)	0.55 (0.40–0.69)	0.56 (0.36–0.76)
2023	0.63 (0.54–0.71)	0.61 (0.53–0.70)	0.58 (0.47–0.70)	0.67 (0.54–0.80)	0.62 (0.39–0.84)	0.63 (0.47–0.79)	0.59 (0.44–0.74)	0.67 (0.49–0.85)
*P*	0.258		0.820	0.168	0.692	0.282	0.688	0.419
**Astigmatism**								
2022	8.87 (8.53–9.22)	8.64 (8.30–8.98)	8.36 (7.89–8.82)	9.43 (8.92–9.94)	7.67 (6.75–8.59)	8.64 (8.04–9.25)	9.18 (8.61–9.76)	9.40 (8.60–10.19)
2023	7.88 (7.58–8.18)	8.06 (7.75–8.36)	7.78 (7.37–8.20)	7.98 (7.55–8.42)	6.68 (5.96–7.41)	7.85 (7.30–8.40)	8.16 (7.62–8.71)	8.25 (7.64–8.86)
*P*	<0.001		0.070	<0.001	0.096	0.057	0.012	0.023
**Anisometropia**								
2022	0.87 (0.76–0.98)	0.87 (0.76–0.98)	0.74 (0.59–0.88)	1.01 (0.83–1.18)	0.85 (0.53–1.16)	0.81 (0.61–1.00)	0.96 (0.76–1.15)	0.81 (0.57–1.05)
2023	0.44 (0.37–0.52)	0.45 (0.38–0.52)	0.45 (0.35–0.56)	0.43 (0.33–0.54)	0.33 (0.16–0.50)	0.45 (0.32–0.59)	0.37 (0.25–0.49)	0.58 (0.42–0.75)
*P*	<0.001		0.001	<0.001	0.002	0.003	<0.001	0.124

**TABLE 3:** Comparison of the prevalence of eye problems in children who received vision screening in different time period of 2022 and 2023, % (95% confidence interval).

Year	Vision screening time		
	Spring (March - May)	Summer (June - July)	*P*
**Eye problems**				
2022	8.75 (8.28–9.22)	10.61 (10.06–11.15)	<0.001
2023	8.20 (7.85–8.55)	9.93 (9.25–10.62)	<0.001
*P*	0.063	0.099	
**Myopia**				
2022	0.84 (0.69–0.99)	0.80 (0.64–0.95)	0.712
2023	0.55 (0.46–0.65)	0.71 (0.52–0.91)	0.121
*P*	0.001	0.513	
**Hyperopia**				
2022	0.52 (0.40–0.63)	0.60 (0.46–0.73)	0.382
2023	0.63 (0.53–0.73)	0.60 (0.43–0.78)	0.777
*P*	0.150	0.948	
**Astigmatism**				
2022	8.01 (7.56–8.46)	9.84 (9.32–10.36)	<0.001
2023	7.50 (7.17–7.83)	9.12 (8.46–9.79)	<0.001
*P*	0.072	0.099	
**Anisometropia**				
2022	0.87 (0.71–1.02)	0.87 (0.71–1.03)	0.981
2023	0.41 (0.33–0.49)	0.55 (0.38–0.72)	0.126
*P*	<0.001	0.012	

**TABLE 4:** Logistic regression analysis on the prevalence of different eye problems.

Covariate	Adjusted odds ratio [*N* (95% confidence interval)]	*P*
**Eye problems**		
Gender	1.058 (0.999–1.119)	0.054
Age	1.070 (1.039–1.102)	<0.001
Vision screening time	1.230 (1.158–1.306)	<0.001
Year	0.928 (0.875–0.984)	0.012
**Myopia**		
Gender	0.842 (0.690–1.026)	0.088
Age	1.395 (1.254–1.552)	<0.001
Vision screening time	1.043 (0.846–1.286)	0.691
Year	0.715 (0.583–0.876)	0.001
**Hyperopia**		
Gender	1.073 (0.867–1.327)	0.516
Age	1.000 (0.896–1.115)	0.995
Vision screening time	1.052 (0.835–1.326)	0.667
Year	1.146 (0.918–1.431)	0.229
**Astigmatism**		
Gender	1.084 (1.022–1.150)	0.007
Age	1.062 (1.030–1.095)	<0.001
Vision screening time	1.241 (1.166–1.321)	<0.001
Year	0.924 (0.869–0.982)	0.011
**Anisometropia**		
Gender	1.198 (0.976–1.471)	0.084
Age	1.073 (0.964–1.195)	0.198
Vision screening time	1.096 (0.883–1.360)	0.405
Year	0.520 (0.418–0.646)	<0.001

The incidence of myopia was 0.82% during the COVID-19 pandemic (in 2022) which was lower than 1%, and was 0.59% after COVID-19 pandemic ended (in 2023) (*P* = 0.001, Figure 1A and Table 2). After standardization, the incidence of myopia was 0.84% in 2022 and 0.60% in 2023. In both male and female children, incidence of myopia was significantly lower in 2023 (male: *P* = 0.003; female: *P* < 0.001; Figures 1B, C and Table 2). In 6-years old children, the incidence of myopia was also significantly lower in 2023 (0.80%), compared to 2022 (1.29%) (*P* = 0.006, Figure 2B and Table 2). In other age groups, myopia incidence were lower in 2023, although the differences were not statistically significant (3-years old: *P* = 0.178; 4-years old: *P* = 0.255; 5-years old: *P* = 0.126; Figure 2B and Table 2). Myopia incidence was also lower in 2023 in children who received vision screening in spring (*P* < 0.001, Table 3). Logistic regression showed that odds of having myopia were significantly larger in older children (adjusted odds ratio = 1.395, *P* < 0.001) and smaller in 2023 (adjusted odds ratio = 0.715, *P* = 0.001, Table 4).

Different from other eye problems, the incidence of hyperopia was slightly higher in 2023 (0.63%) compared to 2022 (*P* = 0.258; Figure 1A and Table 2). The standardized incidence was also 0.55% in 2022 and 0.61% in 2023. After stratification by gender, age or vision screening time, the hyperopia incidence was also slightly higher in 2023 in both male and female children (male: *P* = 0.820; female: *P* = 0.168; Figures 1B, C and Table 2), in children aged 4, 5, or 6 years (4-years old: *P* = 0.282; 5-years old: *P* = 0.688; 6-years old: *P* = 0.419; Figure 2C and Table 2), and in children who received vision screening in spring (*P* = 0.150, Table 3). In children aged 3 years, hyperopia incidence was slightly lower in 2023 (*P* = 0.692; Figure 2C and Table 2).

Among all the eye problems screened, astigmatism showed the highest incidence (8.87% in 2022, and 7.88% in 2023), which was much higher than the other eye problems (see Figure 1A and Table 2). Similar as myopia, prevalence of astigmatism was also significantly lower in 2023 (*P* < 0.001; Figure 1A and Table 2). After standardization, the difference of astigmatism prevalence between 2022 and 2023 was smaller (8.64% in 2022 versus 8.06% in 2023), which may account for the smaller difference of overall eye problems after standardization as mentioned above. Incidence of astigmatism was also lower in 2023 in both male and female children (male: *P* = 0.070; female: *P* < 0.001; Figures 1B, C and Table 2), in different age groups (3-years old: *P* = 0.096; 4-years old: *P* = 0.057; 5-years old: *P* = 0.012; 6-years old: *P* = 0.023; Figure 2D and Table 2), and in children who received vision screening in spring or summer (*P* = 0.072 & 0.099, respectively, Table 3). Logistic regression analysis results indicated that odds of having astigmatism was significantly higher in female children (adjusted odds ratio = 1.084, *P* = 0.007), older children (adjusted odds ratio = 1.062, *P* < 0.001), and when vision screening was performed in summer (adjusted odds ratio = 1.241, *P* < 0.001, Table 4). Odds of having astigmatism were significantly lower in 2023 (adjusted odds ratio = 0.924, *P* = 0.011, Table 4).

Similar as the incidence of myopia or hyperopia, the incidence of anisometropia was lower than 1%. The anisometropia incidence was 0.87% in 2022 and 0.44% in 2023 (*P* < 0.001, Figure 1A and Table 2). After standardization, the anisometropia incidence was 0.87% in 2022 and 0.45% in 2023. After stratification by gender, anisometropia incidence was also significantly lower in 2023 in both male and female children (male: *P* = 0.001; female: *P* < 0.001; Figures 1B, C and Table 2). After stratification by age, all the age groups showed significant lower anisometropia incidence in 2023, except for 6-years-old children (3-years old: *P* = 0.002; 4-years old: *P* = 0.003; 5-years old: *P* < 0.001; 6-years old: *P* = 0.124; Figure 2E and Table 2). After stratification by vision screening time, incidence of anisometropia was also significantly lower in 2023 in children receiving vision screening in spring (*P* < 0.001) and summer (*P* = 0.012, Table 3). Odds of having anisometropia were significantly lower in 2023 (adjusted odds ratio = 0.520, *P* < 0.001, Table 4).

After dividing the vision screening time into spring (March - May) and summer (June - July), the overall incidence of eye problems was significantly higher in summer than in spring in both 2022 and 2023 (*P* < 0.001, Table 3). Further analysis showed that incidence of astigmatism was significantly higher in summer than spring in both 2022 and 2023 (*P* < 0.001, Table 3), and no significant difference was observed between spring and summer in the incidence of myopia, hyperopia, or anisometropia.

## 4 Discussion

During the 3-years COVID-19 pandemic, home confinement measures and more generalized lockdowns were adopted in many countries to slowdown the spreading of the disease. During the COVID-19 pandemic period, myopia progression and increased incidence of astigmatism or anisometropia were observed in school-aged children, possibly due to the dramatically decreased outdoor time and increased screen time (10, 11, 13, 14). In pre-school children, the myopia incidence remained stable during the COVID-19 pandemic, as reported in two previous studies (15, 16). After the ending of COVID-19 pandemic, there is no home confinement and lockdowns, and the social activities have been fully restored. However, studies on the incidence of eye problems after the ending of COVID-19 pandemic in 2023 are still lacking, especially for pre-school children. In this study, by retrospectively collecting vision screening results from regular health examination of pre-school children in Jinniu District, Chengdu, China, we compared the incidence of eye problems of pre-school children aged 3–6 years, including myopia, hyperopia, astigmatism, and anisometropia, during and after the COVID-19 pandemic.

In this study cohort, suspected eye problems were observed in 9.62% of the study cohort during COVID-19 pandemic, and 8.60% after the pandemic. Lower overall incidence of eye problems in 2023 was also observed after standardization by age and vision screening time (9.13% versus 8.78% in 2022 and 2023, respectively), and after stratification (in both male and female children, in all age groups, and in children receiving vision screening in spring or summer). Odds of having eye problems were also significantly lower in 2023. In the four types of eye problems investigated, astigmatism showed the highest incidence (8.87% and 7.88% in 2022 and 2023, respectively, and 8.64% and 8.06% after standardization), which was more than ten times of the incidence of other types of eye problems. As reported in two previous studies from China, the incidence of astigmatism was 33.9% in school-aged children in 2018, and increased to 46.5% and 49.1% in 2020 (10, 11). A previous systemic review and meta-analysis reported a pooled prevalence of 16.5% for astigmatism in Chinese children (22), which is much higher than many other countries [e.g., 3% in India (23), 7.7% in Saudi Arabia (24), 2.2% in Nepal (25), 1% in Ethiopia (26), and 6.7% in Australia (27)]. The reason for the high prevalence of astigmatism in Chinese children could be their ethnicity (more prone to astigmatism) and high academic stress (22). These results indicate that not just in school-aged children, astigmatism is also a major threat of eye health in pre-school children. Similar as myopia, the incidence of astigmatism increases from pre-school children to school-aged children. Currently, most of the studies investigating eye problems were focusing on myopia, and few studies focused on astigmatism. Based on the above-mentioned results, more efforts should be made to understand the underlying mechanism of the high incidence of astigmatism in both pre-school and school-aged children. In addition, efforts should also be made to prevent the occurrence and progression of astigmatism, e.g., earlier screening of astigmatism to allow early intervention, education on parents to increase their awareness on the harm of astigmatism, and encouragement of outdoor activities both in school (between classes) and out of school (after class), in hope of decreasing the incidence of astigmatism in Chinese children in the future.

The incidence of myopia was 0.82% in 2022 and 0.59% in 2023, which were much lower than the previous reports by Yang et al. (10.3%) and by Li et al. (3.3%) (15, 16). These differences in myopia incidence could be partially explained by the different criteria used to define normal/myopia. In the two previous studies, myopia was defined when spherical equivalent <−0.50 D in either eye, while our study used a much stricter criteria (< −3.00 D or < −1.50 D depending on age as described in the Section “2 Materials and methods”). In addition, different from Li et al.’s study which showed limited impact of home confinement on the myopia incidence of pre-school children (16), our study results showed significantly lower myopia incidence after the ending of COVID-19 pandemic, from 0.82% in 2022 to 0.59% in 2023, and significantly lower odds of having myopia in 2023 (adjusted odds ratio = 0.715). A recently-published study by Zontag reported conflicting results that the incidence of uncorrected myopia (≤ −0.5 D) in 5-years old children dramatically increased from 4.9% in year 2013 to 12.6% in year 2023 (28). Similar increases were also observed in the percentage of children with mild myopia (−1.0 D to −0.5 D), moderate myopia (−3.0 D to −1.0 D), or severe myopia (<−3.0 D). Possible explanation of the conflicting results might be different genetic background, length of outdoor activities, and screen time between the two study cohorts. However, since designs of the two studies were both cross-sectional, it is difficult to determine the exact cause of the conflicting results.

Along with these results for myopia, we also observed higher incidence of hyperopia in 2023 (0.63%) compared to 2022 (0.55%) in the study cohort, although the difference was not statistically significant. This result is not surprising since pre-school children normally have slight hyperopia [hyperopia reserves (29)] which gradually disappears after they grow up. After the risk factors for myopia (e.g., home confinement, excessive screen time) were removed after the pandemic, vision of the children tended to return to slight hyperopia, the normal vision of pre-school children. The results of myopia incidence and hyperopia incidence together indicate a hyperopia shift in pre-school children after the ending of COVID-19, possibly due to the increase of outdoor activities and less screen time. The overall hyperopia shift in the pre-school children could have led to the slight increase of hyperopia incidence as we observed.

As shown in the results of this study, the incidence of anisometropia in pre-school children was significantly lower in 2023 (0.44%) compared to 2022 (0.87%). However, literature search revealed that no study has investigated the anisometropia incidence in pre-school children during COVID-19 pandemic. Only a study investigated the anisometropia incidence in school-aged children, and reported a concerning rise in the anisometropia incidence during the COVID-19 pandemic, compared to pre-pandemic period (12).

Our results also showed that overall incidence of eye problems and the incidence of astigmatism were significantly higher in summer than spring in 2022 and 2023, which was not shown in myopia, hyperopia, and anisometropia. These observations might be caused by the progression of astigmatism in these pre-school children in the spring of both 2022 and 2023. A previous study showed myopia progression was faster in spring than summer and autumn (37).

In summary, in this study cohort of pre-school children aged 3–6 years, we observed lower overall incidence of eye problems 2023. Higher incidence of hyperopia and lower incidence of myopia, astigmatism and anisometropia were also observed in 2023. Astigmatism was the most prevalent eye problem in this study cohort, and more attention should be paid to lower the incidence of astigmatism in pre-school children, which could then help lower its incidence in school-aged children and adults. The vision screening in this study followed the criteria released by National Health Commission of the People’s Republic of China, which could be different from the criteria used in other studies (see some of the examples in Table 5). More research efforts are needed to further understand the underlying mechanism of the impacts of COVID-19 pandemic and home confinement on the eye health of pre-school children, which could hopefully help improve the protection of eye health during possible future disease pandemics. Future longitudinal studies with longer follow-ups would help understand whether the change of the incidence of eye problems persists after the ending of COVID-19 pandemic. Future studies could also include behavioral factors (e.g., screen time, reading time, and exposure to sunlight) and investigate the influence of these factors on refractive errors, as well as eye functions (e.g., visual acuity, amblyopia, etc.).

**TABLE 5:** Criteria used in vision examination in different studies.

Study	Criteria
**Myopia**	
Our study	3 ∼ 4 years old: <−3.00 D; 5 ∼ 6 years old: <−1.50 D
Yang et al. (15)	5 ∼ 6 years old: ≤−0.50 D in either eye after cycloplegia
Li Q et al. (16)	3 ∼ 6 years old: ≤−0.50 D in both or either eye
Zontag et al. (28)	5 years old: ≤−0.50 D in either eye
Lan et al. (31)	3 ∼ 6 years old: ≤−0.50 D
Wang X et al. (32)	2 ∼ 6 years old: ≤−0.75 D
Li T et al. (33)	4 ∼ 6 years old: ≤−1.00 D
Al-Rowaily (34)	4 ∼ 8 years old: <−0.50 D
Mehari (35)	0 ∼ 15 years old: ≤−0.50 D in one or both eyes
Zhou et al. (36)	7 ∼ 19 years old: ≤−0.50 D
Wu et al. (37)	4 ∼ 18 years old: ≤−0.50 D in one or both eyes
**Hyperopia**	
Our study	3 ∼ 4 years old: > +4.00 D; 5 ∼ 6 years old: > +3.50 D
Lan et al. (31)	3 ∼ 6 years old: > +2.00 D
Wang X et al. (32)	2 ∼ 6 years old: ≥ +1.75 D
Li T et al. (33)	4 ∼ 6 years old: ≥ +2.00 D
Al-Rowaily (34)	4 ∼ 8 years old: > +2.00 D
Mehari (35)	0 ∼ 15 years old: ≥ +2.00 D in one or both eyes
Zhou et al. (36)	7 ∼ 19 years old: > +0.50 D
Wu et al. (37)	4 ∼ 18 years old: ≥ +0.50 D
**Astigmatism**	
Our study	3 ∼ 4 years old: >2.00 D; 5 ∼ 6 years old: >1.50 D
Lan et al. (31)	3 ∼ 6 years old: >1.50 D
Wang X et al. (32)	2 ∼ 6 years old: ≥1.00 D
Li T et al. (33)	4 ∼ 6 years old: ≥1.00 D
Mehari (35)	0 ∼ 15 years old: ≥0.50 D
Zhou et al. (36)	7 ∼ 19 years old: ≥1.00 D
Wu et al. (37)	4 ∼ 18 years old: ≥0.75 D in either eye
**Anisometropia**	
Our study	3 ∼ 4 years old: differences in hyperopia or myopia > 1.50 D, or difference in astigmatism > 1.00 D; 5 ∼ 6 years old: differences in hyperopia or myopia > 1.50 D, or difference in astigmatism > 1.00 D
Wang X et al. (32)	2 ∼ 6 years old: spherical equivalent difference between the right and left eye ≥ 1.00 D
Zhou et al. (36)	7 ∼ 19 years old: absolute spherical equivalent difference ≥ 1.00 D between eyes
Wu et al. (37)	4 ∼ 18 years old: difference between right eye to left eye in refractive error (spherical error) of ≥1.00 D

## Data Availability

The original contributions presented in this study are included in this article/Supplementary material, further inquiries can be directed to the corresponding author.

## References

[1] World Health Organization [WHO]. *Coronavirus Disease (COVID-19) Pandemic - World Health Organization.* Geneva: World Health Organization (2024).

[2] Ma D Wei S Li SM Yang X Cao K Hu J The impact of study-at-home during the COVID-19 pandemic on myopia progression in Chinese Children. *Frontiers in public health.* (2021) 9:720514. 10.3389/fpubh.2021.720514 35071149 PMC8770940

[3] Ma M Xiong S Zhao S Zheng Z Sun T Li C. COVID-19 home quarantine accelerated the progression of myopia in children aged 7 to 12 Years in China. *Investig Ophthalmol Visual Sci.* (2021) 62:37. 10.1167/iovs.62.10.37 34463719 PMC8411864

[4] Zhang XJ Zhang Y Kam KW Tang F Li Y Ng MPH Prevalence of myopia in children before, during, and after COVID-19 restrictions in Hong Kong. *JAMA Netw Open.* (2023) 6:e234080. 10.1001/jamanetworkopen.2023.4080 36947037 PMC10034576

[5] Wang J Li Y Musch DC Wei N Qi X Ding G Progression of myopia in school-aged children after COVID-19 Home Confinement. *JAMA Ophthalmol.* (2021) 139:293–300. 10.1001/jamaophthalmol.2020.6239 33443542 PMC7809617

[6] Prsova L Halicka J Kozar M Kuderava Z Prso M Jakusova L The prevalence of myopia in school-age children in slovakia and the Covid-19 Pandemic. *Ceska a Slovenska Oftalmologie.* (2023) 79:186–90. 10.31348/2023/24 37567774

[7] Zhang X Cheung SSL Chan HN Zhang Y Wang YM Yip BH Myopia incidence and lifestyle changes among school children during the COVID-19 pandemic: a population-based prospective study. *Br J Ophthalmol.* (2022) 106:1772–8. 10.1136/bjophthalmol-2021-319307 34340973

[8] Kaya P Uzel MM. Development and progression of myopia in children during the COVID-19 pandemic in urban area in Turkey. *Int Ophthalmol.* (2023) 43:3823–9. 10.1007/s10792-023-02824-w 37498447

[9] Ma D Wei S Li SM Yang X Cao K Hu J Progression of myopia in a natural cohort of Chinese children during COVID-19 pandemic. *Graefe’s Arch Clin Exp Ophthalmol.* (2021) 259:2813–20. 10.1007/s00417-021-05305-x 34287693 PMC8294263

[10] Wong SC Kee CS Leung TW. High prevalence of astigmatism in children after school suspension during the COVID-19 pandemic is associated with axial elongation. *Children.* (2022) 9:919. 10.3390/children9060919 35740857 PMC9245603

[11] Liang Y Leung TW Lian JT Kee CS. Significant increase in astigmatism in children after study at home during the COVID-19 lockdown. *Clin Exp Optometry.* (2023) 106:322–30. 10.1080/08164622.2021.2024071 35021950

[12] Huang Y Qiu K Li Y Wang H Zhang M. Temporal trend of anisometropia incidence in Chinese school-aged children before and during the COVID-19 pandemic. *Front Med.* (2024) 11:1322402. 10.3389/fmed.2024.1322402 38410753 PMC10894982

[13] Laan D Tan ETC Huis In Het Veld PI Jellema HM Jenniskens K. Myopia progression in children during home confinement in the COVID-19 pandemic: a systematic review and meta-analysis. *J Optometry.* (2024) 17:100493. 10.1016/j.optom.2023.100493 37879184 PMC10618773

[14] Cyril Kurupp AR Raju A Luthra G Shahbaz M Almatooq H Foucambert P The Impact of the COVID-19 pandemic on myopia progression in children: a systematic review. *Cureus.* (2022) 14:e28444. 10.7759/cureus.28444 36176879 PMC9512310

[15] Yang YC Tsai DC Wang CY Chen YL Shyong MP Hsu NW. The prevalence of myopia remains stable under tighter COVID-19 social restriction in preschoolers receiving a school-based eyecare program. *Acta Ophthalmol.* (2024) 102:e78–85. 10.1111/aos.15680 37144676

[16] Li Q Zhou W Liao Y Chen H Sun Y Wang M Prevalence trend of myopia during the post-COVID-19 epidemic period among preschoolers: a prospective school-based study. *Optometry Vis Sci.* (2023) 100:727–34. 10.1097/OPX.0000000000002069 37678562 PMC11812649

[17] Pan W Lin J Zheng L Lan W Ying G Yang Z Myopia and axial length in school-aged children before, during, and after the COVID-19 lockdown-A population-based study. *Front Public Health.* (2022) 10:992784. 10.3389/fpubh.2022.992784 36589986 PMC9799254

[18] Wang J Han Y Musch DC Li Y Wei N Qi X Evaluation and Follow-up of myopia prevalence among school-aged children subsequent to the COVID-19 home confinement in Feicheng, China. *JAMA Ophthalmol.* (2023) 141:333–40. 10.1001/jamaophthalmol.2022.6506 36821130 PMC9951104

[19] Farook M Venkatramani J Gazzard G Cheng A Tan D Saw SM. Comparisons of the handheld autorefractor, table-mounted autorefractor, and subjective refraction in Singapore adults. *Optometry Vis Sci.* (2005) 82:1066–70. 10.1097/01.opx.0000192344.72997.7c 16357649

[20] Karabulut M Karabulut S Karalezli A. Refractive outcomes of table-mounted and hand-held auto-refractometers in children: an observational cross-sectional study. *BMC Ophthalmol.* (2021) 21:424. 10.1186/s12886-021-02199-5 34879852 PMC8656057

[21] Matta NS Singman EL McCarus C Matta E Silbert DI. Screening for amblyogenic risk factors using the PlusoptiX S04 photoscreener on the indigent population of Honduras. *Ophthalmology.* (2010) 117:1848–50. 10.1016/j.ophtha.2010.01.038 20472292

[22] Tang Y Chen A Zou M Liu Z Young CA Zheng D Prevalence and time trends of refractive error in Chinese children: a systematic review and meta-analysis. *J Glob Health.* (2021) 11:08006. 10.7189/jogh.11.08006 34327000 PMC8285767

[23] Kumar V Soni M Rajagopal V Behera A Gandhi A Shamim MA The prevalence of refractive errors in Indian school children: a systematic review and meta-analysis. *Ophthal Epidemiol.* (2025) 10.1080/09286586.2025.2450346 [Online ahead of print]. 39998413

[24] Al Khathami A Baklola M Alshehri AA Alnasser LH Alshehri RS Salawi MA Prevalence of refractive errors among school-age children and adolescents in saudi arabia: a systematic review and meta-analysis. *Clin Ophthalmol.* (2025) 19:2117–32. 10.2147/OPTH.S539808 40635977 PMC12239900

[25] Bist J Kandel H Paudel N Kaphle D Gyawali R Marasini S Prevalence of refractive errors in Nepalese children and adults: a systematic review with meta-analysis. *Clin Exp Optometry.* (2023) 106:119–32. 10.1080/08164622.2022.2153582 36628479

[26] Atlaw D Shiferaw Z Sahiledengele B Degno S Mamo A Zenbaba D Prevalence of visual impairment due to refractive error among children and adolescents in Ethiopia: a systematic review and meta-analysis. *PLoS One.* (2022) 17:e0271313. 10.1371/journal.pone.0271313 35980970 PMC9387832

[27] Huynh SC Kifley A Rose KA Morgan IG Mitchell P. Astigmatism in 12-year-old Australian children: comparisons with a 6-year-old population. *Investig Ophthalmol Vis Sci.* (2007) 48:73–82. 10.1167/iovs.06-0263 17197519

[28] Zontag N Wygnanski-Jaffe T Bahir D Ben-Zion I. The impact of the COVID 19 pandemic on myopia prevalence in 5 year old Israeli children. *Scientific reports.* (2025) 15(1):14094. 10.1038/s41598-025-98862-8 40269179 PMC12019542

[29] Pu J Fang Y Zhou Z Chen W Hu J Jin S The hyperopia reserve in 3- to 6- years-old preschool children in North China: the Beijing hyperopia reserve research. *BMC Ophthalmol.* (2025) 25:175. 10.1186/s12886-025-04008-9 40197238 PMC11974099

[30] Donovan L Sankaridurg P Ho A Chen X Lin Z Thomas V Myopia progression in Chinese children is slower in summer than in winter. *Optometry Vis Sci.* (2012) 89:1196–202. 10.1097/OPX.0b013e3182640996 22797511 PMC4696401

[31] Lan W Zhao F Lin L Li Z Zeng J Yang Z Refractive errors in 3-6 year-old Chinese children: a very low prevalence of myopia? *PLoS One.* (2013) 8:e78003. 10.1371/journal.pone.0078003 24205064 PMC3813538

[32] Wang X Liu D Feng R Zhao H Wang Q. Refractive error among urban preschool children in Xuzhou, China. *Int J Clin Exp Pathol.* (2014) 7:8922–8. 25674266 PMC4314013

[33] Li T Zhou X Chen X Qi H Gao Q. Refractive Error in Chinese preschool children: the Shanghai study. *Eye Contact Lens.* (2019) 45:182–7. 10.1097/ICL.0000000000000555 30260815 PMC6494031

[34] Al-Rowaily MA. Prevalence of refractive errors among pre-school children at King Abdulaziz Medical City, Riyadh, Saudi Arabia. *Saudi J Ophthalmol.* (2010) 24:45–8. 10.1016/j.sjopt.2010.01.001 23960874 PMC3729549

[35] Mehari ZA. Pattern of childhood ocular morbidity in rural eye hospital, Central Ethiopia. *BMC Ophthalmol.* (2014) 14:50. 10.1186/1471-2415-14-50 24731554 PMC3996137

[36] Zhou Y Cai Q Chen X Huang X Sun Z Song Y Uncorrected refractive errors, visual impairment and need for spectacles among children and adolescents in eastern, China. *PLoS One.* (2025) 20:e0332142. 10.1371/journal.pone.0332142 40956800 PMC12440161

[37] Wu JF Bi HS Wang SM Hu YY Wu H Sun W Refractive error, visual acuity and causes of vision loss in children in Shandong, China. The Shandong Children Eye Study. *PLoS One.* (2013) 8:e82763. 10.1371/journal.pone.0082763 24376575 PMC3871613

